# An optimized reverse β-oxidation pathway to produce selected medium-chain fatty acids in *Saccharomyces cerevisiae*

**DOI:** 10.1186/s13068-023-02317-z

**Published:** 2023-04-26

**Authors:** Fernando Garces Daza, Fabian Haitz, Alice Born, Eckhard Boles

**Affiliations:** Faculty of Biological Sciences, Institute of Molecular Bioscience, Goethe-Universität Frankfurt Am Main, Max-von-Laue-Str.9, 60438 Frankfurt am Main, Germany

**Keywords:** Octanoic acid, Hexanoic acid, *Saccharomyces cerevisiae*, Reverse β-oxidation pathway, Enoyl-CoA hydratase

## Abstract

**Background:**

Medium-chain fatty acids are molecules with applications in different industries and with growing demand. However, the current methods for their extraction are not environmentally sustainable. The reverse β-oxidation pathway is an energy-efficient pathway that produces medium-chain fatty acids in microorganisms, and its use in *Saccharomyces cerevisiae*, a broadly used industrial microorganism, is desired. However, the application of this pathway in this organism has so far either led to low titers or to the predominant production of short-chain fatty acids.

**Results:**

We genetically engineered *Saccharomyces cerevisiae* to produce the medium-chain fatty acids hexanoic and octanoic acid using novel variants of the reverse β-oxidation pathway. We first knocked out glycerolphosphate dehydrogenase *GPD2* in an alcohol dehydrogenases knock-out strain (*△adh1-5*) to increase the NADH availability for the pathway, which significantly increased the production of butyric acid (78 mg/L) and hexanoic acid (2 mg/L) when the pathway was expressed from a plasmid with *BktB* as thiolase. Then, we tested different enzymes for the subsequent pathway reactions: the 3-hydroxyacyl-CoA dehydrogenase PaaH1 increased hexanoic acid production to 33 mg/L, and the expression of enoyl-CoA hydratases Crt2 or Ech was critical to producing octanoic acid, reaching titers of 40 mg/L in both cases. In all cases, Ter from *Treponema denticola* was the preferred trans-enoyl-CoA reductase. The titers of hexanoic acid and octanoic acid were further increased to almost 75 mg/L and 60 mg/L, respectively, when the pathway expression cassette was integrated into the genome and the fermentation was performed in a highly buffered YPD medium. We also co-expressed a butyryl-CoA pathway variant to increase the butyryl-CoA pool and support the chain extension. However, this mainly increased the titers of butyric acid and only slightly increased that of hexanoic acid. Finally, we also tested the deletion of two potential medium-chain acyl-CoA depleting reactions catalyzed by the thioesterase Tes1 and the medium-chain fatty acyl CoA synthase Faa2. However, their deletion did not affect the production titers.

**Conclusions:**

By engineering the NADH metabolism and testing different reverse β-oxidation pathway variants, we extended the product spectrum and obtained the highest titers of octanoic acid and hexanoic acid reported in *S. cerevisiae.* Product toxicity and enzyme specificity must be addressed for the industrial application of the pathway in this organism.

**Supplementary Information:**

The online version contains supplementary material available at 10.1186/s13068-023-02317-z.

## Background

Medium-chain fatty acids (MCFAs) are six to ten hydrocarbon chain long carboxylic acids with applications in a broad range of sectors. In the food industry, MCFA-derived products like methyl hexanoate and ethyl hexanoate, both responsible for the characteristic fragrance of pineapples, are derived from hexanoic acid [62, 69]. In biofuels, 1-octanol can be synthesized from octanoic acid [16]. In polymers and material science, octanoic acid can be used to synthesize polyhydroxy octanoate (PHO), a polymer with rubber-like properties of industrial interest [10, 58], but it can also be used to enhance properties like thermal storage and antibacterial capacity in functional fibers. Currently, MCFAs are either produced via non-sustainable petrochemical routes or extracted directly from palm or coconut tree seeds, representing up to 5 and 10% of the fatty acid content, respectively [43, 61, 65]. However, the extraction of these molecules and the growing demand for vegetable oils come at the price of increasing the extension of monoculture crops, which contributes to deforestation and severe loss in biodiversity [39, 40, 70].

In biotechnology, the short- and medium-chain fatty acids, butyric acid (up to 6 g/L), hexanoic acid (up to 3 g/L) [6, 27, 59] or decanoic acid (up to 277 mg/L) [26] have been produced in engineered *Escherichia coli* via the combination of enzymes from the reverse β-oxidation with specific thioesterases. However, *E. coli* is not a suitable organism for the large-scale production of short- and medium-chain fatty acids due to its low tolerance to organic acids [47, 66], lower robustness and lower resistance to phages compared to other organisms like yeast [41, 50]. Among yeast, *Saccharomyces cerevisiae* is a preferred host for the large scale production of bulk chemicals and organic acids due to its genetic accessibility and the history of successful production of toxic compounds like organic acids, alcohols, aromatic compounds, and volatile esters [15, 18, 29, 41].

**Fig. 1:**
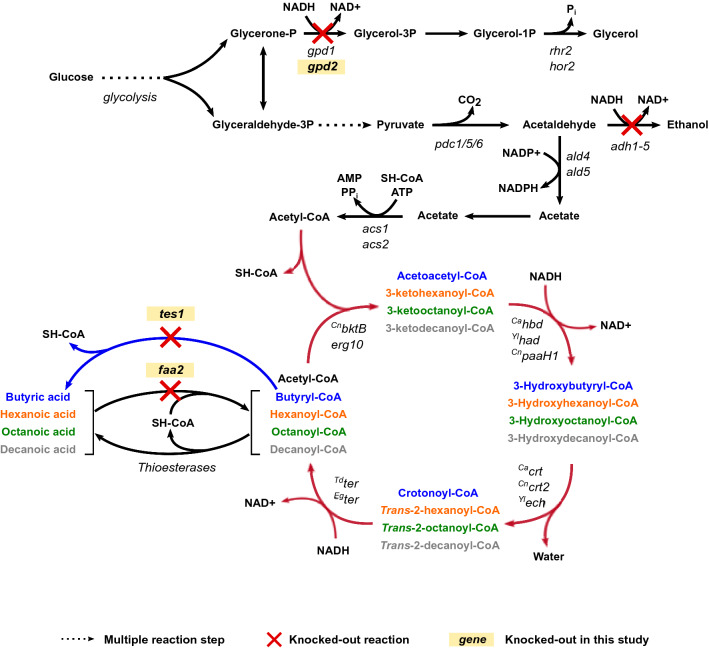
Overview of the reverse β-oxidation pathway and the *S. cerevisiae*’s metabolic modifications used in this study to produce medium-chain fatty acids. The reverse β-oxidation pathway reactions (red arrows) are depicted with the isoenzymes tested in each reaction during this study. The intermediates and products after one cycle (in blue), two cycles (in orange), three cycles (in green), and four cycles (in grey) run of the reverse β-oxidation pathway are shown. The reactions knocked out in the different strains used in this study are displayed with a red cross. The knocked-out reactions generated during this study (*gpd2*, *tes1*, *faa2*) are highlighted in bold. The reaction of *tes1* is shown in blue

The most successful attempts to produce MCFAs by *S. cerevisiae* have so far resulted from directly engineering the fatty acid synthase (FAS), leading to titers of up to 70 mg/L of hexanoic acid and 245 mg/L of octanoic acid [11, 71]. When this approach was combined with engineering the MCFA transporters and performing adaptive laboratory evolution (ALE) to increase tolerance, titers of up to 300 mg/L hexanoic acid, 500 mg/L octanoic acid, and 1.7 g/L decanoic acid were reached [70]. However, the reverse β-oxidation is a more energy-efficient pathway compared to the classical biosynthesis of fatty acids. Unlike the fatty acid biosynthesis, where acetyl-CoA is first converted to the elongation unit malonyl-CoA at the cost of one ATP, the reverse β-oxidation directly uses acetyl-CoA as the elongation unit [9, 18, 38, 50, 58]. The reverse β-oxidation is a cyclical set of four reactions that results in the elongation of an acyl-CoA molecule by two carbon units with each round of the cycle [9] (Fig. 1). It begins with a non-decarboxylative Claisen condensation reaction catalyzed by a thiolase, where acetyl-CoA acts as the donor of two carbon units to an acyl-CoA molecule, generating a β-ketoacyl-CoA. This is later reduced to a 3-hydroxyacyl-CoA by a β-hydroxyacyl-CoA dehydrogenase in a NADH-consuming reaction. The 3-hydroxyacyl-CoA is then dehydrated to an enoyl-CoA by an enoyl-CoA hydratase. Finally, the double bond at the α-carbon of the enoyl-CoA is reduced at the expense of one NADH molecule by a trans-enoyl-CoA reductase, generating an acyl-CoA molecule. This acyl-CoA molecule is available for a new elongation cycle or can be used by a termination enzyme or a different pathway to produce a wide variety of products like alcohols, acids, polyketides, or volatile esters among others [56].

In nature, the reverse β-oxidation pathway is long known in some *Clostridia * spp. like *C. acetobutylicum*, where this pathway is used for the production of butanol [33, 67], or *C. kluyveri*, where it is used to produce butyric acid and hexanoic acid from ethanol and acetate [3, 48]. Recently, it has also been confirmed in *Megasphaera spp.* growing on lactic acid, where it produces hexanoic acid [22, 23]. In addition, some reactions and intermediates of the reverse β-oxidation are also present in other pathways like the polyhydroxyalkanoates (PHA) biosynthesis pathway and the ethylmalonyl-CoA pathway, where thiolase and β-hydroxyacyl-CoA dehydrogenase reactions are key steps [44, 68].

In *S. cerevisiae*, the reverse β-oxidation pathway was first used to produce the four-carbon compound 1-butanol, although at very low titers (2.5 mg/L) [54]. Later, the titers of 1-butanol were significantly improved using a trans-enoyl-CoA reductase from *Treponema denticola* that catalyzes the reduction of enoyl-CoA in an irreversible reaction and by optimizing the NADH and coenzyme A availability, reaching more than 0.85 g/L of 1-butanol [49, 50]. Recently, a new variant of the reverse β-oxidation using the thiolase, BktB, and the β-hydroxyacyl-CoA dehydrogenase, PaaH1, from *Cupriavidus necator* proved to be functional in *S. cerevisiae* to produce hexanoyl-CoA [28], which was used later in this organism as a precursor to synthesize cannabinoids [35]. All in all, the biotechnological application of the reverse β-oxidation pathway works in *S. cerevisiae* but seems limited to two cycles, producing only up to C6-intermediates and products. Extending the length of the products from this pathway in an organism so commonly used in industry like *S. cerevisiae* would accelerate the implementation of sustainable production processes for a previously inaccessible range of products at an industrial scale.

In this study, we aimed to increase the number of cycles when expressing the reverse β-oxidation pathway in the baker’s yeast *S. cerevisiae*. Free fatty acids are easier to measure and quantify than their coenzyme A bound precursors, and *S. cerevisiae* already expresses native thioesterases that hydrolyze medium-chain fatty acyl-CoAs into MCFA [4, 11]. Therefore, we used the production of the short-chain fatty acid butyric acid and of medium-chain fatty acids hexanoic acid (caproic acid), octanoic acid (caprylic acid) and decanoic acid (capric acid) to validate any increase in the number of carbon elongation cycles. For this, we first engineered a *S. cerevisiae* strain with an increased NADH pool, necessary for the successful function of this heterologous pathway, and we studied the effect of deleting the medium-chain fatty acyl-CoA synthase Faa2 and the peroxisomal acyl-CoA thioesterase Tes1, as both reactions could potentially deplete some of the intermediates required for chain elongation. Then, at the pathway level, we screened different enzymes from various organisms at different steps of the reverse β-oxidation pathway, some of them known to catalyze reactions required for the pathway, others only with a putative yet unknown function, and we tested different combinations to fine-tune the length of the products (Fig. 1). Finally, we also assessed the influence of the media composition on the MCFA titers.

## Results

### Thiolase BktB is crucial for medium-chain fatty acid production

We constructed a reverse β-oxidation pathway using *Hbd* and *Crt* from *C. acetobutylicum* as a 3-hydroxyacyl-CoA dehydrogenase and a crotonase, respectively, and *Ter* from *T. denticola* as a trans-2-enoyl-CoA reductase. As a thiolase, we included either *Erg10* from *S. cerevisiae* or *BktB* from *C. necator* to compare their effect on the production of longer chain fatty acids. We compared the two thiolases in *S. cerevisiae* VSY0, a strain lacking the principal alcohol dehydrogenase enzymes (∆*adh1-5*).

**Fig. 2:**
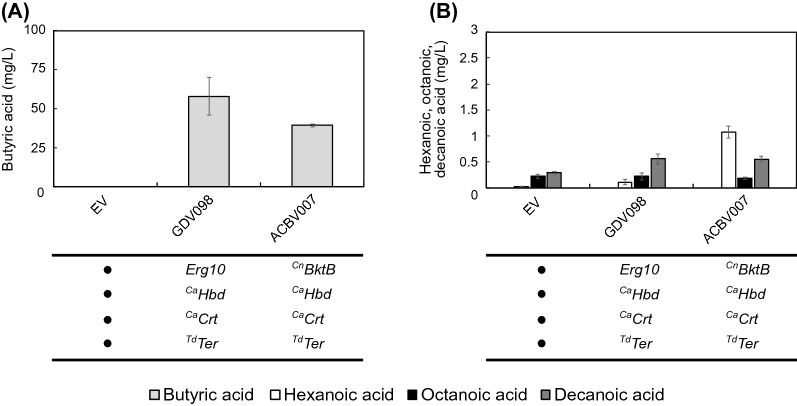
Effect of two different thiolases (Erg10 or BktB) on the production of medium-chain fatty acid. **A** Production of butyric acid (light grey) by VSY0 expressing a reverse β-oxidation pathway with either overexpressed Erg10 as thiolase (GDV098) or BktB as thiolase (ACBV007), or with the empty vector (EV) after 75 h of fermentation in buffered synthetic medium (SM) without uracil. **B** Production of hexanoic (white), octanoic (black) and decanoic acid (dark grey) by VSY0 expressing a reverse β-oxidation pathway with either overexpressed Erg10 as thiolase (GDV098) or BktB as thiolase (ACBV007), or with the empty vector (EV) after 75 h of fermentation in buffered synthetic medium (SM) without uracil. Error bars represent the standard deviation between three independent replicates

Butyric acid, the product of a single cycle of the reverse β-oxidation pathway, was produced when expressing either the *Erg10* pathway variant (GDV098: *Erg10*, ^Ca^*Hbd*, ^Ca^*Crt*, ^Td^*Ter*), or the ^*Cn*^*BktB* pathway variant (ACBV007: ^Cn^*BktB*, ^Ca^*Hbd*, ^Ca^*Crt*, ^Td^*Ter*) (Fig. 2). This indicates that the enzymes selected for the reverse β-oxidation were catalytically active. The *Erg10-*containing pathway (GDV098) supported the production of 58 ± 11,9 mg/L of butyric acid, 47% more than when ^*Cn*^*BktB* (ACBV007) was the preferred thiolase (39,23 ± 0.71 mg/L). The presence of ^*Cn*^*BktB*, on the other hand, slightly increased the production of hexanoic acid (1,07 ± 0,12 mg/L), while the overexpression of *Erg10* did not. Octanoic and decanoic acid were produced in very low amounts, even without a reverse β-oxidation pathway, probably by fatty acid synthases in the cytosol and mitochondria [11]. Regarding decanoic acid, we observed an apparent increase in production with both *Erg10* and ^*Cn*^*BktB*, with 0,559 ± 0,090 mg/L and 0,555 ± 0,052 mg/L, respectively. Altogether, the overall production of MCFAs was deficient (< 2 mg/L), regardless of the thiolase used. Nonetheless, we decided to continue with BktB as the thiolase in further experiments as it produced the highest hexanoic acid titers in this experiment and due to the previously reported ability of this enzyme to produce Medium-chain fatty acyl-CoAs.

### Provision of more NADH increases reverse β-oxidation pathway activity

The availability of NADH is required for the proper function of the reverse β-oxidation and the amount required increases for MCFA synthesis due to the higher number of cycles necessary. We observed that in the absence of alcohol dehydrogenases (VSY0: ∆*adh1-5*), glycerol production increased to regenerate NAD^+^ (Additional file 1: Fig. S1). Therefore, we deleted *GPD2* encoding the primary glycerol 3-phosphate dehydrogenase to reduce the competition for NADH, generating strain GDY15 (∆*adh1-5*, ∆*gpd2*).

**Fig. 3:**
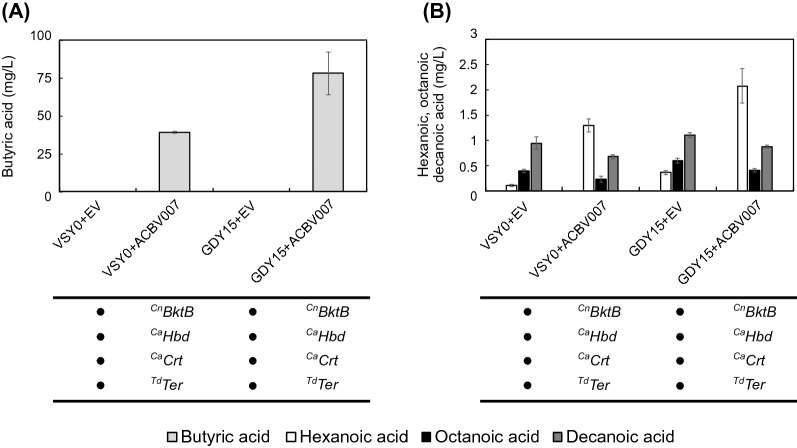
C4–C10 Fatty acid production in a ∆*gpd2* strain. **A** Production of butyric acid (light grey) by VSY0 and GDY15 expressing the reverse β-oxidation pathway (pACB007) or with the empty vector (EV) after 75 h of fermentation in buffered synthetic medium (SM) without uracil. **B** Production of hexanoic (white), octanoic (black) and decanoic acid (dark grey) by VSY0 and GDY15 expressing the reverse β-oxidation pathway (pACB007) or with the empty vector (EV) after 75 h of fermentation in buffered synthetic medium (SM) without uracil. Error bars represent the standard deviation between four independent replicates

We compared GDY15 with VSY0, both transformed with an empty vector or ACBV007 (^Cn^*BktB*, ^Ca^*Hbd*, ^Ca^*Crt*, ^Td^*Ter*), and investigated whether the higher availability of NADH could improve the MCFA production by the reverse β-oxidation pathway (Fig. 3). The deletion of *GPD2* (strain GDY15) reduced, on average, the production of glycerol fivefold (Additional file 2: Table S1). On the other hand, it doubled the production of butyric acid to 78,2 ± 14,18 mg/L (Fig. 3A), and hexanoic acid production was increased by 59%, reaching 2,07 ± 0,34 mg/L (Fig. 3B), indicating a higher flux within the reverse β-oxidation pathway. However, the titers of octanoic and decanoic acid remained very low in all the strains.

Butyric acid was the main product of the pathway also in strain GDY15. This indicates that NADH availability, although pushing the pathway's activity, is not the only factor limiting the production of longer MCFAs.

### The selection of 3-hydroxyacyl-CoA dehydrogenase is crucial for generating MCFAs with reverse β-oxidation

To determine if the reactions following ^*Cn*^*BktB* could be limiting the production of MCFA, we first tested two additional NADH-dependent 3-hydroxyacyl-CoA dehydrogenases: *PaaH1* from *C. necator* and the putative *YALI0C08811* from *Yarrowia lipolytica*, which we will refer to as ^*Yl*^*Had* throughout this study.

**Fig. 4:**
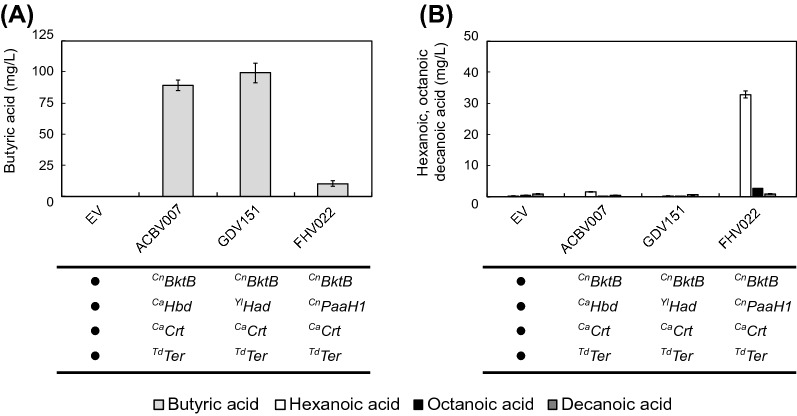
Impact of the β-hydroxyacyl-CoA dehydrogenase selected on the production of MCFA. **A** Production of butyric acid (light grey) by GDY15 expressing the reverse β-oxidation pathway variants (**ACBV007**: ^*Cn*^*BktB*, ^***Ca***^***Hbd***, ^*Ca*^*Crt*, ^*Td*^*Ter*, **GDV151**: ^*Cn*^*BktB*, ^***Yl***^***Had***, ^*Ca*^*Crt*, ^*Td*^*Ter*, **FHV022**: ^Cn^*BktB*, ^**Cn**^***PaaH1***, ^Ca^*Crt*, ^Td^*Ter*) or with the empty vector (EV) after 75 h of fermentation in buffered synthetic medium (SM) without uracil. **B** Production of hexanoic (white), octanoic (black) and decanoic acid (dark grey) by GDY15 expressing the reverse β-oxidation pathway variants (ACBV007, GDV151, FHV022) or with the empty vector (EV) after 75 h of fermentation in buffered synthetic medium (SM) without uracil. Error bars represent the standard deviation between four independent replicates

We transformed GDY15 with the plasmids ACBV007 (^Cn^*BktB*, ^Ca^*Hbd*, ^Ca^*Crt*, ^Td^*Ter*), FHV022 (^Cn^*BktB*, ^*Cn*^*PaaH1*, ^Ca^*Crt*, ^Td^*Ter*), GDV151 (^Cn^*BktB*, ^Yl^*Had*, ^Ca^*Crt*, ^Td^*Ter*) and compared the impact of each 3-hydroxyacyl-CoA dehydrogenase on butyric acid (Fig. 4A) and MCFA production (Fig. 4B). While the variants expressing ^Ca^*Hbd* (ACBV007) and ^Yl^*Had* (GDV151) produce mainly butyric acid (89,25 ± 4,03 and 99,2 ± 7,85 mg/L, respectively), in the variant expressing ^Cn^*PaaH1* (FHV022) the main product is hexanoic acid in considerable amounts (32,88 ± 1,09 mg/L). In this variant, not only the titer of butyric acid (10,35 ± 2,10 mg/L) is decreased compared to ^Ca^*Hbd* (ACBV007) and ^Yl^*Had* (GDV151), but also the octanoic acid titer (2,68 ± 0.04 mg/L) is higher than in those variants (*p*-value < 0.0001), none of which reached a titer of 1 mg/L.

Regarding ^Ca^*Hbd* and ^Yl^*Had*, although the butyric acid titers are not significantly different between them, we observed a trend towards higher butyric acid titers with ^Yl^*Had*. Also, while ACBV007 (^Ca^*Hbd*) produced 1,57 ± 0,13 mg/L of hexanoic acid, GDV151 (^Yl^*Had*) did not produce significant amounts of any MCFA.

### The enoyl-CoA hydratase reaction also controls the fatty acid chain length

The choice of ^Cn^PaaH1 as an adequate enzyme at the 3-hydroxyacyl-CoA dehydrogenase reaction increased the overall MCFA production up to 20-fold. However, the chain length seemed to be limited to hexanoic acid. Therefore, we wanted to test whether the choice of enoyl-CoA hydratase could also have an impact on the chain length of the MCFA. To assess this, we initially tested one additional enoyl-CoA hydratase, *Crt2* from *C. necator*, first described by [51].

**Fig. 5:**
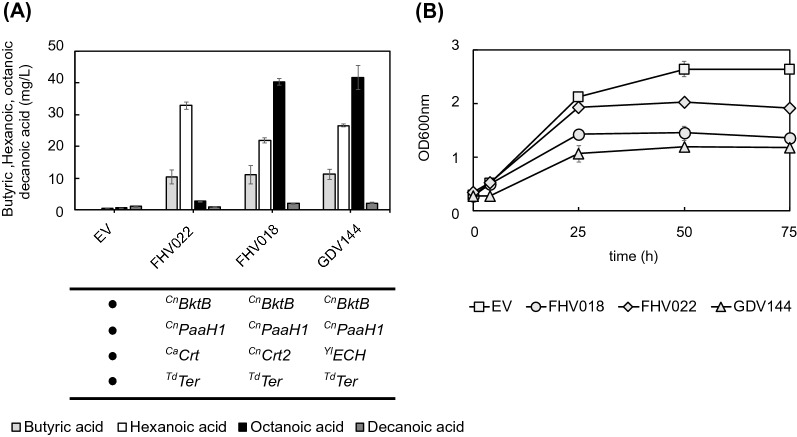
Growth and MCFA production with different enoyl-CoA. **A** Production of butyric (light grey), hexanoic (white), octanoic (black) and decanoic acid (dark grey) by GDY15 expressing the reverse β-oxidation pathway variants (**FHV022**: ^Cn^*BktB*, ^Cn^*PaaH1*, ^**Ca**^***Crt***, ^*Td*^*Ter*), (**FHV018**: ^*Cn*^*BktB*, ^Cn^*PaaH1*, ^***Cn***^***Crt2***, ^*Td*^*Ter*), (**GDV144**: ^*Cn*^*BktB*, ^Cn^*PaaH1*, ^***Yl***^***ECH****, *^*Td*^*Ter*) or with the empty vector (EV) **B** Growth of GDY15 with EV (white square), FHV018 (white circle), FHV022 (white diamond) or GDY144 (white triangle) over 75 h in buffered synthetic medium (SM) without uracil. Error bars represent the standard deviation between three independent replicates

We transformed GDY15 with the plasmids FHV018 (^Cn^*BktB*, ^Cn^*PaaH1*, ^Cn^*Crt2*, ^Td^*Ter*), FHV022 (^Cn^*BktB*, ^Cn^*PaaH1*, ^Ca^*Crt*, ^Td^*Ter*) or an empty vector (EV) and compared the impact of each enoyl-CoA hydratase on the production of butyric acid and MCFA. The presence of ^Cn^*Crt2* (FHV018) as enoyl-CoA hydratase did not change the final titers of butyric acid, but it almost doubled the overall production of MCFA compared to when ^Ca^*Crt* (FHV022) was present (Fig. 5A, Additional file 2: Table S2). The production of octanoic acid increased 15-fold (40,26 ± 1,05 mg/L). On the other hand, hexanoic acid titers still reached 21,88 ± 0,78 mg/L with FHV018 (^Cn^*Crt2*) but remained lower compared to FHV022 (^Ca^*Crt*) (32,88 ± 1,09 mg/L). Although still at relatively low titers, decanoic acid increased twofold in the presence of ^Cn^Crt2.

Analysis in PROSITE showed that ^Cn^*Crt2* has an amino acid signature (PS00166) typically found in the enoyl-CoA hydratase/isomerase family [7]. We screened for enzymes with this motif in *S. cerevisiae* and some known oleaginous yeast [30] and performed a Protein BLAST of the best candidates against ^Cn^*Crt2*. As a result, *YALI0B10406p*, a putative mitochondrial enoyl-CoA hydratase from *Y. lipolytica* which will be referred to as ^*Yl*^*ECH* throughout this study, resulted as the most promising candidate and was tested for MCFA production as plasmid GDV144 (^Cn^*BktB*, ^Cn^*PaaH1*, ^Yl^*ECH*, ^Td^*Ter*). When expressing this pathway variant, titers of hexanoic acid and octanoic acid reached 26,57 ± 0,39 mg/L and 41,66 ± 3,81 mg/L, respectively. Compared to FHV018 (^Cn^*Crt2*), the GDV144 (^*Yl*^*ECH*) variant produced higher titers of hexanoic acid; therefore, the hexanoic acid to octanoic acid ratio increased almost 20% in this strain. No significant changes in butyric acid production were observed in this strain compared to FHV018 (^Cn^*Crt2*) or FHV022 (^Ca^*Crt*), and the production of decanoic acid was also not improved.

We also observed that the final OD_600nm_ decreased with hexanoic acid production (FHV022) and decreased further with octanoic as the main product (FHV018 and GDV144), suggesting a growth inhibitory effect proportional to the chain length of the main fatty acid produced (Fig. 5B). In addition, we observed that, in the octanoic acid producing variants, the expression of ^*Yl*^*ECH* (GDV144) results in a slight growth impairment compared to ^Cn^*Crt2* (FHV018).

### The selection of an appropriate trans-enoyl-CoA reductase determines the product titers

The *trans*-enoyl-CoA reductase reaction was a bottleneck in the reverse β-oxidation pathway when producing *n*-butanol [49]. Therefore, we tested one additional *trans*-enoyl-CoA reductase from the microalgae *Euglena gracilis*, which will be referred to as ^Eg^Ter, lacking its mitochondrial targeting sequence. This enzyme was first described by Inui et al. [20] as part of a mitochondrial form of anaerobic fatty acid synthesis in *E. gracilis*, and later characterized by Hoffmeister et al. [19]. ^Eg^Ter has a reported higher specificity for *trans*-2-hexenoyl-CoA and *trans*-2-octenoyl-CoA than for crotonoyl-CoA [20], which makes it a suitable candidate to improve the MCFA production.

**Fig. 6:**
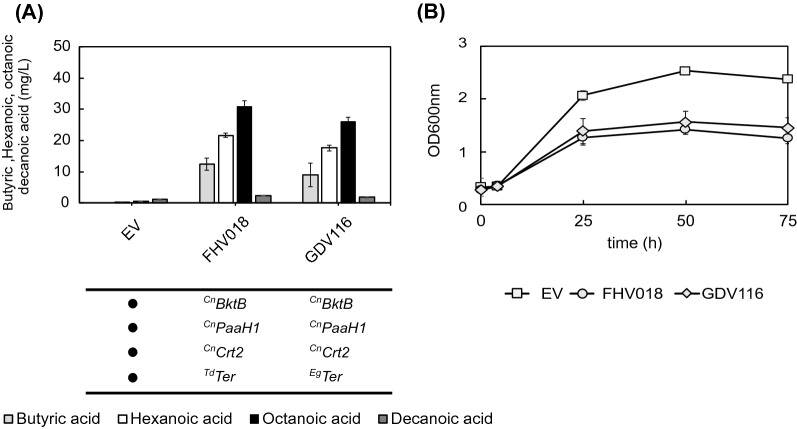
Growth and MCFA production with different *trans*-enoyl-CoA reductases.** A** Production of butyric (light grey), hexanoic (white), octanoic (black) and decanoic acid (dark grey) by GDY15 expressing the reverse β-oxidation pathway variants (**FHV018**: ^*Cn*^*BktB*, ^Cn^*PaaH1*, ^*Cn*^*Crt2*, ^*Td*^**Ter**,  **GDV116**: ^Cn^*BktB*, ^Cn^*PaaH1*, ^Cn^*Crt2*, ^Eg^**Ter**) or with the empty vector (EV) **B** Growth of GDY15 with EV (white square), FHV018 (white circle), or GDV116 (white diamond) over 75 h in synthetic medium (SM) without uracil. Error bars represent the standard deviation between three independent replicates

We transformed GDY15 with the plasmid GDV116 (^Cn^*BktB*, ^Cn^*PaaH1*, ^Cn^*Crt2*, ^Eg^*Ter*) and compared the production of butyric acid and MCFA against FHV018 (^Cn^*BktB*, ^Cn^*PaaH1*, ^Cn^*Crt2*, ^Td^*Ter*) and an empty vector control after 75 h of fermentation in SM medium. The production pattern did not change, with octanoic acid as the main MCFA with both *trans*-enoyl-CoA reductases, followed by hexanoic acid (Fig. 6A). However, the expression of ^Eg^*Ter* (GDV116) significantly decreased the overall production of MCFA (45,57 ± 1,59 mg/L) compared to ^Td^*Ter* (FHV018) (54,89 ± 1,89 mg/L). Butyric acid production did not significantly differ between *trans*-enoyl-CoA reductases, although we also see a trend of lower titers with ^Eg^*Ter* (GDV116).The production of significant titers of MCFA results, again, in impaired growth compared to the empty vector control (Fig. 6B). However, the growth with both plasmids FHV018 and GDV116 is similar, suggesting that the higher MCFA production by FHV018 (^Td^*Ter*) is not growth-related,but that ^Td^*Ter* is a better enzyme for this reaction. When we compare the MCFA yield on biomass (Y_MCFA/X_) and the yield on glucose (Y_MCFA/S_) (Additional file 2: Table S3) of each clone, we observed that GDY15 expressing ^Td^*Ter* as *trans*-2-enoyl-CoA reductase had a 40% and 54% higher yield on biomass and glucose, respectively, compared to ^Eg^*Ter*.

Finally, the yield of glycerol on biomass (Y_GLY/X_) and on glucose (Y_GLY/S_) was higher with ^Eg^*Ter* (GDV116), 13% and 40% respectively, than with ^Td^*Ter* (FHV018). This might indicate that NADH re-oxidation via the ^Eg^Ter-containing pathway variant was insufficient, leading to increased glycerol production through the remaining Gpd1.

### The deletions of FAA2 and TES1 do not improve MCFA production

**Fig. 7:**
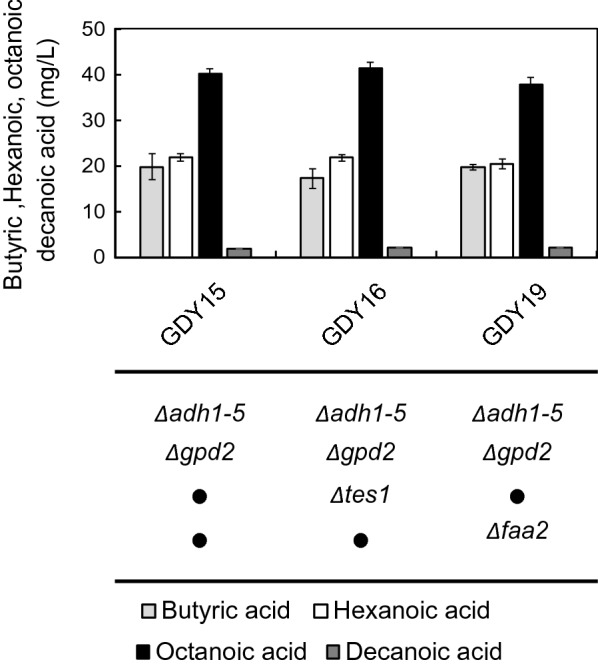
Effect of deleting potential competing reactions on the production of MCFA. Production of butyric (light grey), hexanoic (white), octanoic (black) and decanoic acid (dark grey) in GDY15, GDY16 (*∆tes1*) and GDY19 (*∆faa2*) expressing the reverse β-oxidation pathway (FHV018: ^*Cn*^*BktB*, ^Cn^*PaaH1*, ^*Cn*^*Crt2*, ^*Td*^*Ter*). Error bars represent the standard deviation between three independent replicates

Competing reactions could still hinder the production of MCFAs. To increase the production of free MCFA, we deleted *TES1* or *FAA2* in strain GDY15, generating strains GDY16 (∆*adh1-5*, ∆*gpd2*, ∆*tes1*) and GDY19 (∆*adh1-5*, ∆*gpd2*, ∆*faa2*). However, deleting these genes did not significantly increase MCFA nor reduce butyric acid titers or yields compared to their parental GDY15 strain (Fig. 7, Additional file 2: Table S4).

### Increasing butyryl-CoA availability boosts hexanoic acid production

Butyryl-CoA is the product of a single cycle of the reverse β-oxidation pathway. It is key to producing hexanoyl-CoA, a precursor of hexanoic acid that can be further elongated to octanoyl-CoA, precursor of octanoic acid. Therefore, we tested whether increasing the first round of the reverse β-oxidation pathway could further improve MCFA production. In GDY15 we combined GDV124 (^*Cn*^*BktB, *^*Cn*^*PaaH1, *^*Cn*^*Crt2, *^*Td*^*Ter*) with GDV098 (*Erg10, *^*Ca*^*Hbd, *^*Ca*^*Crt, *^*Td*^*Ter*) or an empty vector (EV) control.

**Fig. 8:**
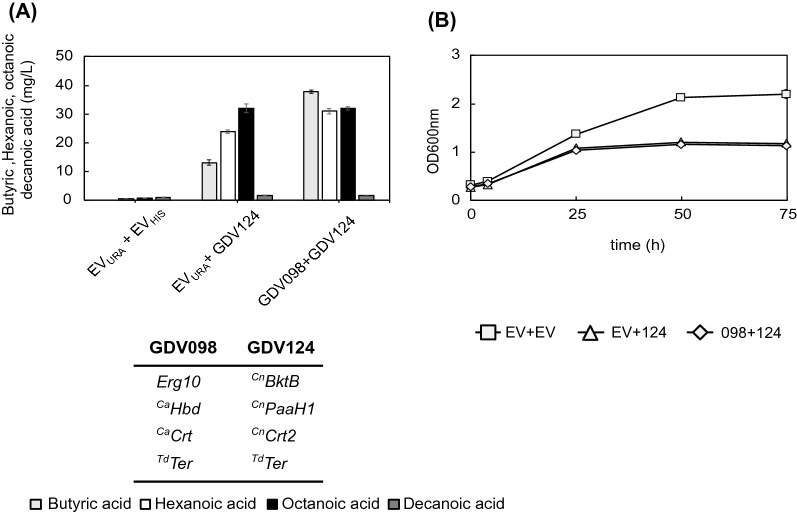
Effect of increasing butyryl-CoA availability on MCFA production. **A** Production of butyric (light grey), hexanoic (white), octanoic (black) and decanoic acid (dark grey) in GDY15 expressing the MCFA producing pathway alone (GDV124: ^*Cn*^*BktB, *^*Cn*^*PaaH1, *^*Cn*^*Crt2, *^*Td*^*Ter*), in combination with GDV098 (*Erg10, *^*Ca*^*Hbd, *^*Ca*^*Crt, *^*Td*^*Ter*) or with empty vector (EV_URA_ + EV_HIS_) controls. **B** Growth of GDY15 with EV (white square), EV_URA_ + GDV124 (white triangle, or GDV098 + GDV124 (white diamond) over 75 h in synthetic medium (SM) without uracil or histidine. Error bars represent the standard deviation between three independent replicates.

The addition of a butyryl-CoA producing pathway increased butyric acid titers almost threefold (GDV098 + GDV124) (37,79 ± 0,61 mg/L) compared to when the MCFA producing pathway operates alone (EV_URA_ + GDV124) (13,09 ± 0,93 mg/L) (Fig. 8A). Hexanoic acid production also increased 23% when expressing GDV098 (31,04 ± 0,96 mg/L), probably due to higher availability of butyryl-CoA. However, neither octanoic nor decanoic acid titers changed significantly with the additional expression of the butyryl-CoA producing. No significant butyric acid or MCFA were detected in the absence of any reverse β-oxidation pathway variant.

As observed in previous experiments, the growth of the MCFAs-producing strains was impaired compared to that of the non-producing strains (Fig. 8B). Interestingly, the higher butyric acid production in the strain expressing both GDV098 and GDV124 did not compromise the growth compared to the strain only expressing the MCFA-producing pathway.

### Fermentation medium and buffering effect determines MCFA production

As observed above, an increased chain length in MCFAs decreased growth. In addition, strategies to increase the chain length improved hexanoic acid yield on biomass (Y_HEX/X_), but not those of octanoic (Y_OCT/X_) or decanoic acid (Y_DEC/X_) (Additional file 2: Table S5). We compared growth and MCFA production in buffered synthetic medium (SM) against an equally buffered (20 mM phosphate) YPD medium to test whether these limitations arise from the medium composition used during fermentations. In addition, we also tested if an increased buffering capacity could improve MCFA production. For that, we also tested YPD with 100 mM phosphate concentration (YPD-100 mM). Since no auxotrophic markers can be used in YPD and to avoid the addition of antibiotics to the fermentation medium, we genomically integrated the hexanoic acid (with ^Ca^Crt) and the octanoic acid (with ^Cn^Crt2 or ^Yl^ECH) producing pathway cassettes in strain GDY15, generating strains GDY27 (^Ca^Crt), GDY28 (^Cn^Crt2) and GDY29 (^Yl^ECH). We fermented GDY15 and the producing strains in different media up to 75 h and compared MCFA and butyric acid production after 50 h and 75 h.

**Fig. 9:**
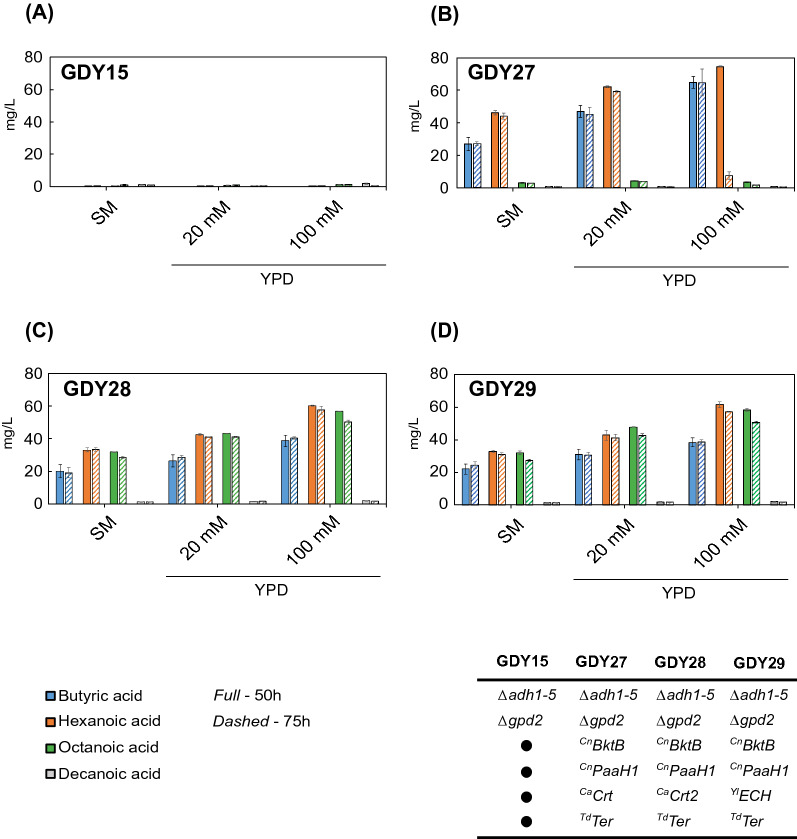
MCFA production in strains with integrated reverse β-oxidation pathway variants in different cultivation media. Production of butyric (blue), hexanoic (orange), octanoic (green) and decanoic acid (dark grey) by strains GDY15 (**A**), GDY27 (**B**), GDY28 (**C**) and GDY29 (**D**) after 50 h (*Full*) or 75 h (*Dashed*) of fermentation in synthetic medium (SM), YPD with 20 mM phosphate buffer or YPD with 100 mM phosphate buffer. Error bars represent the standard deviation between three independent replicates

Overall, the production titers of butyric acid, hexanoic acid and octanoic acid peaked at 50 h in the three different fermentation media tested (Fig. 9). Also, production titers were higher in YPD-based medium than in SM and increased further with increased buffering. This pattern was observed in all the strains except for GDY15 (Fig. 9A). In this strain, there was no butyric acid production and all MCFA were produced at titers below 2 mg/L.

After 50 h in YPD-20 mM, the butyric acid, hexanoic acid and octanoic acid production titers in GDY27 (Fig. 9B) reached 46,97 ± 3,71 mg/L, 62,17 ± 0,6 mg/L and 4,27 ± 0,04 mg/L, respectively, representing a 75%, 35% and 45% increase compared to titers in SM. Butyric and hexanoic acid titers were further increased in YPD-100 mM, reaching 64,74 ± 3,57 mg/L and 74,58 ± 0,39 mg/L, respectively. In the case of hexanoic acid and to our knowledge, these are the highest titers produced in *S. cerevisiae* using the reverse β-oxidation pathway. In this strain, octanoic acid titers were slightly reduced (3,29 ± 0,21 mg/L) in YPD-100 mM.

In the octanoic acid producing strains, GDY28 and GDY29, octanoic acid production increased by 36% in GDY28 (43,04 ± 0,28 mg/L) and by 50% in GDY29 (47,98 ± 0,15 mg/L) in YPD-20 mM compared to SM (Fig. 9C, D). In these two strains, butyric acid and hexanoic acid increased in YPD-20 mM by 30%, on average, compared to fermentation in SM, with butyric acid and hexanoic acid reaching 26,31 ± 4,31 mg/L and 42,53 ± 0,93 mg/L in GDY28, respectively, and 31,03 ± 3,38 mg/L (butyric acid) and 42,92 ± 3,23 mg/L (hexanoic acid) in GDY29. As in the case of GDY27, the titers of butyric, hexanoic and octanoic acid increased in both strains when cultivated in the more buffered YPD-100 mM medium. Here, butyric acid and hexanoic acid titers reached 38,75 ± 2,22 mg/L and 60.23 ± 0.78 mg/L in GDY28, respectively, and 38,46 ± 2,65 mg/L and 61.67 ± 1.71 mg/L in GDY29. Regarding the production of octanoic acid by these two strains in YPD-100 mM, we produced 56.88 ± 0.41 mg/L in GDY28 and 58.31 ± 1.06 mg/L in GDY29, a 30% and a 20% improvement to the octanoic acid titers observed in YPD-20 mM and, to our knowledge, the highest titers of octanoic acid produced in yeast via the reverse β-oxidation.

After 75 h of fermentation, we observed a slight decrease in the final titers of butyric acid, hexanoic acid and octanoic acid in all strains. Interestingly, the only exception was GDY27, where hexanoic acid was almost completely consumed after 75 h in YPD-100 mM (7,51 ± 2.23 mg/L) (Fig. 9B). We observed this phenomenon only in this strain and in separate experiments when cultivated in YPD-100 mM (data not shown).

**Fig. 10:**
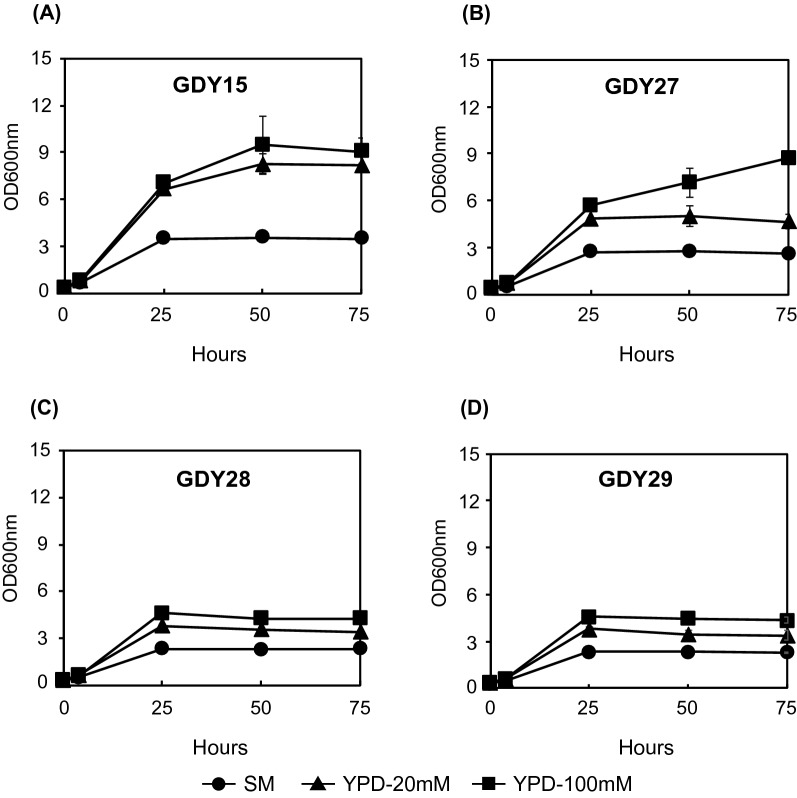
Growth of *S. cerevisiae* strains with integrated reverse β-oxidation pathway variants in different cultivation media. Growth (OD_600nm_) of strain GDY15 (**A**), GDY27 (**B**), GDY28 (**C**) and GDY29 (**D**) in synthetic medium (SM) (filled circle) YPD with 20 mM phosphate buffer (filled triangle) and YPD with 100 mM phosphate buffer (filled square) over 75 h. Error bars represent the standard deviation between three independent replicates

The composition of the fermentation medium and the phosphate buffer concentration also significantly influenced the growth of different strains, their metabolite consumption and production pattern (Fig. 10, Additional file 1: Fig. S2, S3, Additional file 2: Table S6). While in SM glucose was never completely consumed in any of the strains, switching to YPD-20 mM increased the overall glucose consumption of the strain by 60% and led to glucose being depleted from the fermentation medium in GDY15 and GDY27 already after 50 h, and in GDY28 and GDY29 after 75 h. Fermentation in YPD-20 mM also more than doubled (2.4-fold) the final OD_600nm_ of the control strain GDY15 (Fig. 10A), increased it by 80% for GDY27 (Fig. 10B) and by 46% in both octanoic acid producers compared to fermentation in SM (Fig. 10C, D).

Further increasing the buffering capacity of the medium (YPD-100 mM) resulted in the total consumption of glucose after 50 h of fermentation in all the strains (Additional file 1: Fig. S2). Fermentation in YPD-100 mM also boosted the growth of all strains (except for GDY15), which was particularly noticeable in strain GDY27, where the final OD_600nm_ increased by 87% compared to when grown in YPD-20 mM. In the case of the octanoic acid producing strains GDY28 and GDY29, final OD_600nm_ increased by 25% and 28%, respectively, compared to those in YPD-20 mM.

## Discussion

In this study, we modified the ethanol, glycerol and redox metabolism of a *S. cerevisiae* strain and identified optimal combinations of enzymes to produce butyric, hexanoic, and octanoic acid via different variants of a reverse β-oxidation pathway.

We generated a strain with blocked competing pathways and an increased NADH availability, a co-factor required by the heterologous pathway. This strategy was previously successful both in *E. coli* and *S. cerevisiae*, where deletion of main NADH consuming pathways led to an increased production of *n*-butanol or hexanol [49, 52]. We observed that our initial strain VSY0 (∆*adh1-5*) produced significant glycerol titers (Additional file 1: Fig. S1A). In *S. cerevisiae*, glycerol production starts with an NADH-consuming reduction of dihydroxyacetone phosphate to glycerol 3-phosphate [42], catalyzed by Gpd2 to prevent NADH accumulation in anaerobic conditions [1, 2]. Therefore, we knocked out *GPD2* to avoid this NADH pool being used for the formation of glycerol. This led to an increased productivity of the reverse β-oxidation pathway (Fig. 3). Similar strategies consisting of deleting both *GPD1* and *GPD2* genes to prevent competition for NADH have resulted in increased final titers of iso-butanol [64].

We identified specific combinations of enzymes optimal for butyric, hexanoic and octanoic acid production. The presence of Erg10, ^Ca^Hbd or ^Yl^Had limited the production to butyric acid. The thiolase, Erg10, catalyzes the formation of acetoacetyl-CoA from two acetyl-CoA molecules in the mevalonate pathway, critical for the formation of isoprenoids in eukaryotes [17]. Erg10 was previously selected as the best thiolase to produce 1-butanol in *S. cerevisiae* [50]. However, no MCFAs titers were determined in that study. Here, we show that its thiolase activity is limited to the condensation of two acetyl-CoA molecules. ^Ca^Hbd is part of the conserved 1-butanol production pathway found in different clostridia species [33] and, therefore, specific towards the 4-carbon long 3-hydroxybutyryl-CoA. Surprisingly, a pathway with ^Ca^Hbd was reported to produce up to 101 mg/mL of hexanoic acid in *K. marxianus* grown in YPD [5]. However, in *S. cerevisiae* our main product was butyric acid. ^Yl^Had is a putative mitochondrial 3-hydroxyacyl-CoA dehydrogenase from *Y. lipolytica* [60]. To the best of our knowledge, ^Yl^Had had only been tested once to produce butanol and MCFA in *S. cerevisiae* [34] where it produced up to 8 mg/mL of octanoic acid. However, we did not detect significant titers of products longer than butyric acid with this enzyme in our study. A possible explanation for this difference might be the impact of the medium-chain specific thioesterase (FatB1 from *Candida parapsilosis*) used in the experiment by Lian and Zhao [34].

The presence of ^Cn^BktB and ^Cn^PaaH1 was critical for producing hexanoic and octanoic acid. We selected ^Cn^BktB because it has been reported to catalyze the condensation of acetyl-CoA with butyryl-CoA or hexanoyl-CoA [6, 8, 35], and we confirmed this by producing hexanoic and octanoic acid. In the specific case of acetyl-CoA condensation with hexanoyl-CoA, we observed this activity for the first time in *S. cerevisiae*. In the screening, we included ^Cn^PaaH1, a 3-hydroxyacyl-CoA dehydrogenase with a reported crystal structure (PDB:4PZC) [24], because of its known affinity for 3-oxohexanoyl-CoAs [28, 36]. Here, we show for the first time that, besides reducing 3-oxohexanoyl-CoA, it can also catalyze the reduction of 3-oxooctanoyl-CoA that leads to the production of octanoic acid.

Selecting an adequate enoyl-CoA hydratase was essential to tune the pattern of MCFAs produced. Our study shows that both ^Cn^Crt2 and ^Yl^ECH are suitable enoyl-CoA hydratases for producing MCFAs up to octanoic acid. Based on previous studies, the substrate preference of ^Yl^ECH was limited to 3-hydroxybutyryl-CoA when used in a modified reverse β-oxidation pathway in *S. cerevisiae* [34]. In the case of ^Cn^Crt2, its activity on 3-hydroxyhexanoyl-CoA was known after its overexpression in a modified *C. necator* strain increased the production of poly((*R*)-3-hydroxybutyrate-co-(*R*)-3-hydroxyhexanoate), a polymer made partially of hexanoyl-CoA [68]. Here, we show for the first time that ^Yl^ECH can also use 3-hydroxyhexanoyl-CoA as substrate, and that both enzymes can use 3-hydroxyoctanoyl-CoA, leading to octanoic acid as the main product. The clostridial ^Ca^Crt limited the production to hexanoic acid, confirming its previously observed activity towards 3-hydroxyhexanoyl-CoA, which has been exploited to produce hexanoyl-CoA, a key precursor for cannabinoid synthesis [35, 55, 57] and also to produce hexanoic acid in *E. coli* [59]. Since under the same conditions no significant octanoic or decanoic acids were produced in the presence of ^Ca^Crt compared to ^Cn^Crt2 or ^Yl^ECH, this suggests that ^Ca^Crt has no affinity towards 3-hydroxyacyl-CoAs longer than 6-carbons.

The enzyme variants selected for the reactions preceding the enoyl-CoA reductase seem to limit the production to octanoic acid, as no relevant titers of decanoic acid were obtained. This hampered a comparison between the activities towards longer chain substrates of the *trans*-enoyl-2-CoA reductases tested (^Eg^Ter or ^Td^Ter). However, we observed a higher yield of MCFA production when ^Td^Ter was present, suggesting a higher catalytic activity with this enzyme under the conditions tested.

To discard the possibility of ^Cn^BktB cleaving decanoyl-CoA, given that the condensation reaction catalyzed by thiolases is only thermodynamically favored at high substrate concentrations [9], we tested the co-expression of a MCFA producing pathway variant with a butyryl-CoA producing pathway to boost MCFA. With this, we expected to increase the concentrations of medium chain acyl-CoAs upstream to decanoyl-CoA. However, this only led to butyric acid as the main product and only increased hexanoic acid titers (Fig. 8A). In a previous work by Kim and Gonzalez [26], the combination of ^Cn^BktB with ^Eg^Ter, two enzymes also used in our study, led to titers close to 100 mg/L of decanoic acid in *E. coli* [26]. However, a dual 3-hydroxyacyl-CoA dehydrogenase/ enoyl-CoA hydratase (fadB) was used in that study, and it is possible that the 3-hydroxyacyl-CoA dehydrogenase and enoyl-CoA hydratases combinations used in our study have no affinity for 10-carbon 3-hydroxyacyl-CoAs or 3-oxoacyl-CoAs.

We also tested the deletions of endogenous thioesterase *TES1* and medium chain acyl-CoA synthase *FAA2* to prevent the produced MCFA being consumed via β-oxidation. The short-chain acyl-CoA thioesterases Tes1 is known to target butyryl-CoA in *S. cerevisiae* [37]. This could lead to an increased butyric acid production that would compromise the chain elongation capacity of the pathway. Faa2, on the other hand, is a medium-chain fatty acyl-CoA synthetase that activates medium-chain free fatty acids in the peroxisomes, where they diffuse, and this leads to their degradation via β-oxidation [31]. Nonetheless, we did not observe any significant change in titers or the pattern of MCFA production in either case (Fig. 7). Tes1 is located in the peroxisomes of *S. cerevisiae*, while the reverse β-oxidation enzymes were expressed in the cytosol, making the impact of its deletion harder to assess due to pre-existing limited access of this thioesterase to the acyl-CoA intermediates of the pathway. As a comparison, in *E. coli* the deletion of TesB, a cytosolic thioesterase closely related to Tes1 [21, 37], led to a significant increase in the C6-C10 MCFA fraction produced by the expression of the reverse β-oxidation in the cytosol of this organism [25]. Regarding Faa2, while the deletion of *FAA2* is reported to increase the production of medium-chain fatty acids [31] and medium-chain fatty alcohols in *S. cerevisiae* [16], it had no impact on MCFA titers under the conditions tested in this study.

We initially screened the different combinations of genes for the reverse β-oxidation on centromeric plasmids. However, we observed increased titers upon integrating the best enzyme combinations in the genome (Fig. 5 and Fig. 9). Improved expression after genomic integration has been observed previously in *S. cerevisiae* [49, 50]. It could be explained by a lower plasmid burden, particularly when using big (> 10 kb) plasmids like the ones in this study, but also by the higher expression variability between cells observed when heterologous genes are expressed from plasmids compared to when expressed from the genome [32]. Besides chromosomal integration, the composition of the fermentation medium also played an essential role in the titers obtained, as we achieved the highest titers of MCFA in YPD with 100 mM phosphate buffer at pH 6.3 (Fig. 9). Increasing the buffering capacity in the medium for MCFA production was previously effective in *S. cerevisiae* strains engineered to produce octanoic acid via a modified FAS enzyme [4, 11]. We hypothesize that increasing buffering capacity at pH 6.3 prevents the anionic forms of butyric, hexanoic and octanoic acid (pKa’s around 4.8–4.9) to re-enter the cell through passive diffusion. This way, the cell only has to metabolize the intracellularly produced MCFAs, where a portion of them will not be charged due to a lower cytosolic pH than the medium [46], and could therefore exit the cell by passive diffusion. The remaining cytosolic charged fraction of the MCFAs should be either consumed in the β-oxidation or possibly secreted via Pdr12, an ABC transporter involved in the adaptation to weak acid stress in yeast [45].

This study aimed to extend the chain length of MCFA produced in *S. cerevisiae* via reverse β-oxidation further than hexanoic acid. We identified new enzyme combinations to produce octanoic acid in *S. cerevisiae*. However, the 3-hydroxyacyl-CoA dehydrogenase and enoyl-CoA hydratases selected here seem to limit further elongation of the products. Furthermore, butyric acid was present in both hexanoic acid and octanoic acid-producing strains, but this might be derived from the reaction of the endogenous Erg10 and additionally ^Cn^BktB. Recently, Vogeli et al. [59] reported high titers of hexanoic acid with minimal amounts of butyric acid as a side product in a modified *E. coli* strain combining a thiolase, ThlA, and a 3-hydroxyacyl-CoA dehydrogenase, Hbd, from *C. autoethanogenum* with ^Ca^Crt and ^Td^Ter. In that study, butyric acid titers were further reduced by overexpressing putative C6-C10 specific thioesterase TesA. Even though a different organism and a different hydroxybutyryl-CoA dehydrogenase was used in their study, further investigation on alternative reverse β-oxidation enzymes and thioesterases is needed to improve product specificity in *S. cerevisiae*. In addition, octanoic acid titers are below those reported by engineering FAS [11] and both pathways seem to be limited by the toxicity of their products. Therefore, preventing toxicity is the next goal towards industrial MCFA production.

## Conclusions

In this study, we achieved the highest titers of hexanoic acid and octanoic acid reported in *S. cerevisiae* using the reverse β-oxidation pathway. The expression of this pathway in this yeast had previously resulted in the production of only up to 6-carbon products. To extend the number of cycles of this pathway in this organism, we tackled competing reactions by deleting *GPD2* and then separately *FAA2* and *TES1*. However, only the deletion of *GPD2* resulted in increasing titers. We also studied the effect of different enzymes in different steps of the pathway and established a reverse β-oxidation pathway that resulted in the production of octanoic acid as the main product. Furthermore, we showed for the first time that Crt2 from *C. necator* and the putative enoyl-CoA hydratase ECH from *Y. lipolytica* prefer 8-carbon long enoyl-CoA over shorter molecules. Finally, our results also show that production is correlated to the buffering capacity of the fermentation medium, which suggests that product toxicity is an issue to be tackled if MCFA are to be produced at higher titers using the reverse β-oxidation.

## Materials and methods

### Strains and cultivation media

Yeast strains used in this study are listed in Table 1. *S. cerevisiae* strains were grown in YPD medium containing 10 g/L yeast extract, 20 g/L peptone, 20 g/L glucose and 20 mM or 100 mM of potassium dihydrogen phosphate or in Synthetic medium (SM) containing 1.7 g/L Yeast nitrogen base (without aminoacids), 5 g/L ammonium sulfate, 20 mM of potassium dihydrogen phosphate, 20 g/L glucose and supplemented with 3.6 g/L leucine, 2.4 g/L tryptophan, 2.4 g/L histidine and 1.2 g/L uracil. *S. cerevisiae* expressing plasmids were grown in Synthetic medium (SM) with or without histidine or uracil supplementation. All yeast cultivation media were adjusted to pH 6.3. The bacteria *E. coli* DH10β was used for plasmid construction and subcloning and was grown in Lysogeny Broth (LB) containing 5 g/L of yeast extract, 10 g/L of Trypton, 5 g/L of sodium chloride and supplemented with 50 µg/mL of Kanamycin.

**Table 1:** List of yeast strains and plasmids used in this study

Strain	Genotype	Reference
CENPK2-1c	*MATa; ura3-52; trp1-289; leu2-3_112*; *his3 Δ1; MAL2-8C; SUC2*	EUROSCARF
SHY34	*MATa; ura3-52; trp1-289; leu2-3_112; his3 Δ1; MAL2-8C; SUC2, Δfas1, Δfas2, Δfaa2*	[63]
VSY0	*MATa; ura3-52; trp1-289; leu2-3_112; his3 Δ1; MAL2-8C; SUC2 adh1::loxP adh2Δ::LEU2 adh3::loxP adh4Δ::loxP adh5::loxP*	[49, 50]
GDY15	*MATa; ura3-52; trp1-289; leu2-3_112; his3 Δ1; MAL2-8C; SUC2 adh1::loxP adh2Δ::LEU2 adh3::loxP adh4Δ::loxP adh5::loxP gpd2Δ*	This study
GDY16	*MATa; ura3-52; trp1-289; leu2-3_112; his3 Δ1; MAL2-8C; SUC2 adh1::loxP adh2Δ::LEU2 adh3::loxP adh4Δ::loxP adh5::loxP gpd2Δ tes1Δ*	This study
GDY19	*MATa; ura3-52; trp1-289; leu2-3_112; his3 Δ1; MAL2-8C; SUC2 adh1::loxP adh2Δ::LEU2 adh3::loxP adh4Δ::loxP adh5::loxP gpd2Δ faa2Δ*	This study
GDY27	GDY15 *Δura3*::pHHF2-^*Cn*^*bktB*-tENO2, pCCW12-^*Cn*^*paaH1*-tIDP1, pENO2-^*Ca*^*crt*-tPGK1, pTDH3- ^*Td*^*ter* -tADH1, *KanMX*	This study
GDY28	GDY15 *Δura3*:: pHHF2- ^*cn*^*bktB* -tENO2, pCCW12-^*Cn*^*paaH1*-tIDP1, pENO2-^*Cn*^*crt2*-tPGK1, pTDH3- ^*Td*^*ter* -tADH1, *KanMX*	This study
GDY29	GDY15 *Δura3*:: pHHF2-*cnBktB*-tENO2, pCCW12-^Cn^*paaH1*-tIDP1, pENO2-^*Yl*^*ech*-tPGK1, pTDH3- ^*Td*^*ter* -tADH1, *KanMX*	This study

Plasmid	Characteristics	Reference
ACBV007	*URA3*, CEN6ARS4, *Kanamycin R*, ColE1, pHHF2- ^*cn*^*bktB* -tENO2, pCCW12- ^*Ca*^*hbd* -tIDP1, pENO2-^*Ca*^*crt*-tPGK1, pTDH3- ^*Td*^*ter* -tADH1	This study
GDV098	*URA3*, CEN6ARS4, *Kanamycin R*, ColE1, pPGK1-e*rg10*-tENO2, pCCW12- ^*Ca*^*hbd* -tIDP1, pENO2- ^*Ca*^*crt* -tPGK1, pTDH3- ^*Td*^*ter* -tADH1	This study
FHV022	*URA3*, CEN6ARS4, *Kanamycin R*, ColE1, pHHF2- ^*cn*^*bktB* -tENO2, pCCW12- ^*Cn*^*paaH1*-tIDP1, pENO2-^*Ca*^*crt*-tPGK1, pTDH3- ^*Td*^*ter* -tADH1	This study
GDV151	*URA3*, CEN6ARS4, *Kanamycin R*, ColE1, pHHF2- ^*cn*^*bktB* -tENO2, pCCW12- ^*Yl*^*had*-tIDP1, pENO2-^*Ca*^*crt*-tPGK1, pTDH3- ^*Td*^*ter* -tADH1	This study
FHV018	*URA3*, CEN6ARS4, *Kanamycin R*, ColE1, pHHF2- ^*cn*^*bktB* -tENO2, pCCW12-^*Cn*^*paaH1*-tIDP1, pENO2-^*Cn*^*crt2*-tPGK1, pTDH3- ^*Td*^*ter* -tADH1	This study
GDV144	*URA3*, CEN6ARS4, *Kanamycin R*, ColE1, pHHF2- ^*cn*^*bktB* -tENO2, pCCW12- ^*Cn*^*paaH1*-tIDP1, pENO2- ^*Yl*^*ech* -tPGK1, pTDH3- ^*Td*^*ter* -tADH1	This study
GDV116	*URA3*, CEN6ARS4, *Kanamycin R*, ColE1, pHHF2- ^*cn*^*bktB* -tENO2, pCCW12- ^*Cn*^*paaH1*-tIDP1, pENO2- ^*Cn*^*crt2*-tPGK1, pTDH3- ^*Eg*^*ter* -tADH1	This study
GDV124	*HIS3*, CEN6ARS4, *Kanamycin R*, ColE1, pHHF2- ^*cn*^*bktB* -tENO2, pCCW12- ^*Cn*^*paaH1*-tIDP1, pENO2- ^*Cn*^*crt2*-tPGK1, pTDH3- ^*Td*^*ter* -tADH1	This study
pVS6	*Ampicillin R*, ColE1, GPD2 5ʹ pHXT7-*erg10*-tVMA16, pPGK1- ^*Ca*^*hbd* -tEFM1, pTPI1-*CaCRT*-tYHI9, pPYK1-^*Td*^*ter* -tIDP1, pADH1-*CaADHE2*-tRPL3, pTDH3-*EcEutE*-tRPL41B, *KanMX*, GPD2 3ʹ	[49]
pVS1	*Ampicillin R*, ColE1, GPD2 5ʹ pHXT7-*erg10*-tVMA16, pPGK1-^*Ca*^*hbd*-tEFM1, pTPI1-*CaCRT*-tYHI9, loxP, *KanMX*, loxP, pPYK1- ^*Eg*^*ter* -tIDP1, pADH1-*CaADHE2*-tRPL3, GPD2 3ʹ	[49]
pRCCK_GD01	*KanMX*,2µ, *Ampicillin R*, ColE1, *Cas9*, gRNA for GPD2 deletion	This study
FHV016	*KanMX*,2µ, *Ampicillin R*, ColE1, *Cas9*, gRNA for TES1 deletion	This study
pRCCK_SH06	*CloNat*,2µ, *Ampicillin R*, ColE1, *Cas9*, gRNA for FAA2 deletion	Sandra Born (Boles lab)
GDV143	*Kanamycin R*, ColE1, URA3 5ʹ UTR, pHHF2- ^*cn*^*bktB* -tENO2, pCCW12- ^*Cn*^*paaH1*-tIDP1, pENO2- ^*Cn*^*crt2*-tPGK1, pTDH3- ^*Td*^*ter* -tADH1, *KanMX*, URA3 3ʹ UTR	This study
GDV149	*Kanamycin R*, ColE1, URA3 5ʹ UTR, pHHF2- ^*cn*^*bktB* -tENO2, pCCW12- ^*Cn*^*paaH1*-tIDP1, pENO2-^*Ca*^*crt*-tPGK1, pTDH3- ^*Td*^*ter* -tADH1, *KanMX*, URA3 3ʹ UTR	This study
GDV143	*Kanamycin R*, ColE1, URA3 5' UTR, pHHF2- ^*cn*^*bktB* -tENO2, pCCW12- ^*Cn*^*paaH1*-tIDP1, pENO2- ^*Cn*^*crt2*-tPGK1, pTDH3- ^*Td*^*ter* -tADH1, *KanMX*, URA3 3' UTR	This study
GDV150	*Kanamycin R*, ColE1, URA3 5ʹ UTR, pHHF2- ^*cn*^*bktB* -tENO2, pCCW12- ^*Cn*^*paaH1*-tIDP1, pENO2- ^*Yl*^*ech* -tPGK1, pTDH3- ^*Td*^*ter* -tADH1, *KanMX*, URA3 3ʹ UTR	This study
LBGV022	*URA3*, CEN6ARS4, *Kanamycin R*, ColE1	Leonardo J. Beltran (Boles lab)
LBGV024	*HIS3*, CEN6ARS4, *Kanamycin R*, ColE1	Leonardo J. Beltran (Boles lab)

Geneticin and Nourseothricin resistance cassettes are indicated by ‘KanMX’ and ‘CloNat’, respectively. Histidine and uracil auxotrophic complementation is also indicated by ‘HIS3’ and ‘URA3’, respectively. The origin of replication regions ‘2µ’ and ‘CENARS’ are also indicated for non-integrative plasmids. Genes from Clostridium acetobutylicum (Ca), Cupriavidus necator (Cn), Euglena gracilis (Eg) or Yarrowia lipolytica (Yl) are indicated as superscript prefixes. Acetoacetyl-CoA thiolase (erg10), β-ketoacyl-CoA thiolase (BktB) 3-hydroxyacyl-CoA dehydrogenase (paaH1, had), 3-hydroxybutyryl-CoA dehydrogenase (hbd), crotonase/ enoyl-CoA hydratase (crt, crt2, ech), trans-2-enoyl-CoA reductase (ter)

### Cultivation of microorganisms

Yeast strains were pre-cultured in 10 mL of appropriate cultivation medium (YPD or SM HIS^−^ and/ or URA^−^) in agitation (180 rpm) and at 30ºC. Pre-cultures were harvested in exponential phase, washed twice in sterile water and 100 mL Erlenmeyer flasks containing 40 mL of appropriate medium were inoculated at a starting OD_600nm_ of 0.3. Fermentations were carried over 75 h at 30 °C and 180 rpm in semi-anaerobic conditions and in triplicates or quadruplicates. Samples for metabolite analysis in HPLC were taken at 0, 4, 25, 50 and 75 h. Samples for MCFA analysis were taken at 50 and 75 h.

### Plasmid and strain construction

BktB (UniProt ID: Q0KBP1), PaaH1 (UniProt ID: Q0KEY8), CRT2 (UniProt ID:Q0K6J5) from *C. necator*, HAD (UniProt ID:Q6CCJ7) and ECH (UniProt ID: Q6CF43) from *Y. lipolytica* were codon optimized using the JCat software [14], and the genes were ordered as gene blocks from Twist Bioscience. The mitochondrial targeting sequences from HAD and ECH were omitted. ERG10 (*S. cerevisiae*) (UniProt ID: P41338), HBD (*C. acetobutylicum*) (UniProt ID: P52041), CRT (*C.* *acetobutylicum*) (UniProt ID: P52046) and TER (*T. denticola*) (UniProt ID: Q73Q47) were amplified by PCR from pVS6, TER (*E. gracilis*) (UniProt ID: Q5EU90) was PCR amplified from pVS1. All the amino acid sequences of the proteins used in this study can be found in Additional file 2: Table S8. All primers used in this study can be found in Additional file 2: Table S7. All ordered or PCR amplified DNA fragments were first cloned into pYTK001 for further subcloning using Golden Gate DNA assembly (GGA). A list of all the GGA plasmids used in this study can be found in Additional file 2: Table S9. The different parts used to build the GGA plasmids are those reported in [32].

Scarless deletions of *GPD2*, *TES1* and *FAA2* were performed using the CRISPR/Cas9 system described in [12]. For *GPD2* and *TES1* deletions, 80 bp donor DNA fragments with 40 bp homology upstream and downstream of each gene were co-transformed with plasmids pRCCK_GD01 and FHV016, respectively. For *FAA2* deletion, the plasmid pRCCK_SH06 was co-transformed with a PCR amplified *FAA2* region of a *∆faa2-ko* strain (SHY34), which was used as donor DNA. The crRNAs required for recognition by the Cas9 endonucleases in FHV016, pRCCK_GD01 and pRCCK_SH06 were designed using the gRNA designer online tool from ATUM (https://www.atum.bio/catalog/vectors/grna-design). Strains GDY27, GDY28, GDY29 derive from the integration of the respective expression cassettes from plasmids GDV149, GDV143, and GDV150 in strain GDY15. The integration cassettes were first linearized by an overnight digestion with NotI, the restriction enzyme was then deactivated and the whole digestion reaction was transformed in GDY15. The linearized integration cassettes contained 500 bp homologous regions upstream and downstream of the *URA3* loci. Yeast transformations were performed according to [13]. Positive clones were selected on YPD-agar plates with 200 µg/mL Geneticin. All deletions were confirmed by PCR.

### Medium-chain fatty acid extraction and derivatization

To extract the free fatty acids (FFA) produced in the fermentation, 13 mL of each culture were centrifuged (3000 rcf, 10 min, RT) and the pelleted cell fraction discarded. Then, 200 µg of heptanoic acid were added as internal standard to 10 mL of each supernatant and mixed with 1 mL of a 1 M HCl and 2.5 mL of a methanol/chloroform solution (1:1). The solution was vigorously shaken for 5 min and then centrifuged (3000 rcf, 10 min, RT). The chloroform fraction was transferred to a 1.5 mL Eppendorf tube and evaporated overnight.

For fatty acid methylation, both the samples from the FFA extraction and standard samples containing 5, 25, 50, 100 and 200 mg/L of hexanoic, octanoic and decanoic acid (Sigma–Aldrich) were dissolved in 200 µL of toluene, mixed with 1.5 mL of methanol and 300 µL of an 8.0% (w/v) HCl solution, vortexed vigorously and incubated at 100 °C for 3 h to generate fatty acid methyl ester (FAME). Then, samples were cooled at 4 °C for 15 min and 1 mL of water and 1 mL hexane were added. The mixture was briefly shaken in a vortex and after a clear separation of the organic and aqueous phase, the organic phase was transferred to a GC vial.

### Fatty acid methyl esters analysis in GC-FID

GC analyses for detection and quantification of MCFA were carried out on a Perkin Elmer Clarus 400 instrument (Perkin Elmer, Germany) equipped with an Elite 5MS capillary column (30 m × 0.25 mm, film thickness 1.00 µm, Perkin Elmer, Germany) and a flame ionization detector (Perkin Elmer, Germany). 1 μL of the sample was analyzed after split injection (1:10) and helium was used as carrier gas (90 kPa). For quantification of FAMEs, the temperatures of the injector and detector were 250 and 300 °C, respectively. The following temperature program was used: 50 °C for 5 min; increase of 10 °C/min to 120 °C and hold for 5 min; increase of 15 °C/min to 220 °C and hold for 10 min; increase of 20 °C/min to 300 °C and hold for 5 min. Medium-chain FAMEs were identified and quantified by comparison to FAMEs in standard samples.

### Metabolite analysis in HPLC

The glucose, glycerol, acetic acid, ethanol and butyric acid produced in the fermentations were analysed by HPLC in a Dionex UltiMate 3000 (ThermoScientific) system equipped with a Coregel 87H3 (Concise Separations) column and a refractive index (RI) detector (Thermo Shodex RI-101). 1 mL of sample was centrifuged (12 min, 4 °C, 15,000 g), the pellet discarded and the supernatant filtered with a 0.22 μm nylon-membrane filter and transferred to a new 1.5 mL tube. 450 μL of the filtered supernatant were then mixed with 50 µL of 50% (w/v) 5-sulfosalicylic acid, centrifuged (10 min, RT, 15,000 g) and transferred to a HPLC vial with a screw cap. The HPLC was operated at 40 °C with 5 mM sulfuric acid and a constant flow rate of 0.4 mL/min. Glucose, glycerol, acetic acid, ethanol and butyric acid were identified and quantified by comparison to standard samples ranging from 0.1 to 20 g/L for glucose, glycerol and ethanol, and by comparison to standard samples ranging from 0.01 to 5 g/L for acetic acid and butyric acid.

### PROSITE analysis

The complete amino acid sequence of ^Cn^Crt2 was scanned in ScanProsite to find the protein signature in this enzyme. Then, a list of all the UniProtKB entries matching the protein signature PS00166 (Enoyl-CoA hydratase/Isomerase) was retrieved and filtered by hits in *Cryptococcus curvatus*, *Lipomyces lipofer*, *Rhodosporidium toruloides*, *Rhodotorula glutinis*, *S. cerevisiae* and *Y. lipolytica*. The presence of mitochondrial targeting sequences (MTS) in these hits was assessed with MitoProt II (https://ihg.helmholtz-muenchen.de/ihg/mitoprot.html), and if an MTS was detected, it was removed from the sequence for further analysis. The complete PROSITE, MitoProt II and BLAST analysis is shown in Additional file 2: Table S10.

### Statistical analysis

A minimum of three independent replicates are used in all the experiments described in this study. The two-tailed Student’s *t*-test for independent samples is used to assess the statistical significance between strains. For the experiment presented in Fig. 8, a two-tailed *t*-test for paired samples is used to determine differences between different medium compositions for each strain after 50 h of fermentation. In both cases, the significance level is 5%. All statistical analysis were performed using GraphPad Prism. The complete statistical analysis is shown in Additional file 2: Table S11.

## Supplementary Information

**Additional file 1: Figure S1–S3**. Growth, glycerol and ethanol production in *S. cerevisiae* strains (WT, VSY0, GDY15) (Figure S1). Glucose consumption (S2) and acetic acid production (S3) by *S. cerevisiae* strains with integrated reverse β-oxidation pathway variants in different cultivation media.

**Additional file 2: Table S1–S11**. Contains additional tables to expand and support the Results of this study (yields on biomass, yield on substrate, glucose consumption, etc.) as well as the protein sequences of the enzymes, PROSITE analysis, statistical analysis, the list of primers and a list of additional plasmids used to build the final plasmids used in this study.

## Data Availability

All data and materials generated or analyzed during this study are included in this published article and its Additional information files or will be made available by the authors upon reasonable request.

## Abbreviations

ATP: Adenosine triphosphate
ABC transporter: ATP-binding cassette transporter
BLAST: Basic local alignment search tool
BktB: β-Ketothiolase
Ca: *Clostridium acetobutylicum*
CloNat: Nourseothricin resistance gene
Cn: *Cupriavidus necator* (*Ralstonia eutropha*)
CoA: Coenzyme A
CRISPR: Clustered Regularly Interspaced Short Palindromic Repeats
crRNA: CRISPR RNA
DNA: Deoxyribonucleic acid
Eg: *Euglena gracilis*
FAME: Fatty acid methyl ester
FAS: Fatty acid synthase
FFA: Free fatty acids
GC: Gas chromatography
GC-FID: Gas chromatography with flame ionization detector
GGA: Golden Gate DNA assembly
Gpd: Glycerol 3-phosphate dehydrogenase
HCl: Hydrochloric acid
HIS3: Imidazoleglycerol-phosphate dehydratase gene (histidine selection marker)
HPLC: High-performance liquid chromatography
KanMX: Geneticin resistance gene
MCFA: Medium-chain fatty acids
MTS: Mitochondrial targeting sequence
NA: Not available
NAD^+^: Nicotinamide adenine dinucleotide (oxidized form)
NADH: Nicotinamide adenine dinucleotide (reduced form)
PCR: Polymerase chain reaction
Rcf: Relative centrifugal force
RNA: Ribonucleic acid
RT: Room temperature
SM: Synthetic medium
SM URA^−^: Synthetic medium without uracil
SM HIS^−^: Synthetic medium without histidine
Td: Treponema denticola
URA3: Orotidine 5-phosphate decarboxylase (uracil selection marker)
Y_BUT/S_: Yield of butyric acid on glucose
Y_BUT/X_: Yield of butyric acid on biomass
Y_HEX/S_: Yield of hexanoic acid on glucose
Y_HEX/X_: Yield of hexanoic acid on biomass
Y_OCT/S_: Yield of octanoic acid on glucose
Y_OCT/X_: Yield of octanoic acid on biomass
Y_DEC/S_: Yield of decanoic acid on glucose
Y_DEC/X_: Yield of decanoic acid on biomass
Y_MCFA/S_: Yield of medium-chain fatty acids on glucose
Y_MCFA/X_: Yield of medium-chain fatty acids on biomass
Y_X/S_: Yield of biomass on glucose
Yl: *Yarrowia lipolytica*
YPD: Yeast Peptone Dextrose cultivation medium
YPD-20 mM: YPD medium with 20 mM phosphate buffer
YPD-100 mM: YPD medium with 100 mM phosphate buffer
