# Supplementary material for: An optimized reverse β-oxidation pathway to produce selected medium-chain fatty acids in Saccharomyces cerevisiae
Source: Biotechnol Biofuels Bioprod. 2023 Apr 26;16:71. doi: 10.1186/s13068-023-02317-z (PMC10134560; doi:10.1186/s13068-023-02317-z)
Supplement: Supplementary file 1 — Additional file 1: Figure S1–S3. Growth, glycerol and ethanol production in S. cerevisiae strains (WT, VSY0, GDY15) (Figure S1). Glucose consumption (S2) and acetic acid production (S3) by S. cerevisiae strains with integrated reverse β-oxidation pathway variants in different cultivation media. [file 13068_2023_2317_MOESM1_ESM.docx]

# **Additional file 1**

An optimized reverse β-oxidation pathway to produce selected medium-chain fatty acids in *Saccharomyces cerevisiae*

JF Garcés Daza^1^, F Haitz^1^, A Born^1^, E Boles^1 *^

^1^ Faculty of Biological Sciences, Institute of Molecular Bioscience, Goethe-Universität Frankfurt am Main, Max-von-Laue-Str.9, 60438, Frankfurt am Main, Germany

^*^ Corresponding author

E-mail address: e.boles@bio.uni-frankfurt.de


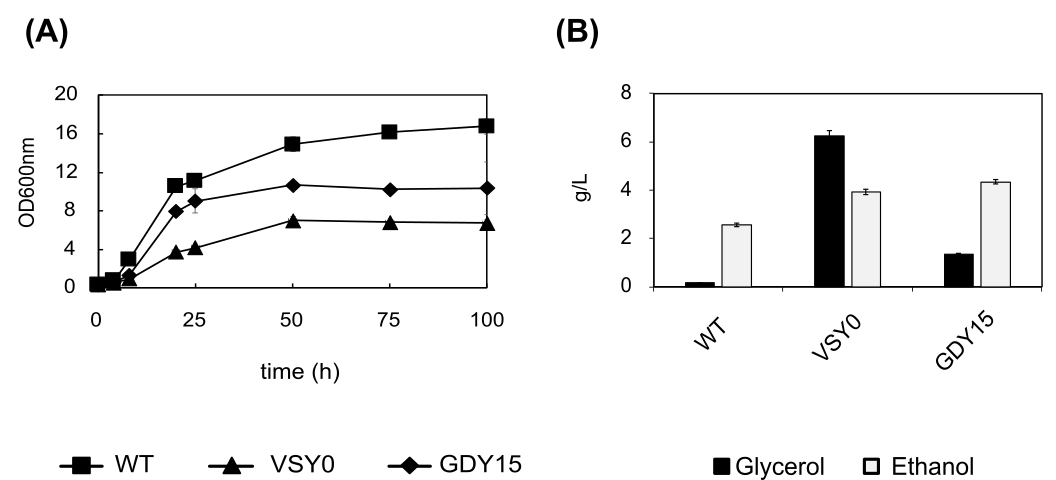


**Figure S1. Growth, glycerol and ethanol production in *S. cerevisiae* strains.** A) Growth of WT (*filled square*, ■), *adh-ko* strain VSY0 (*filled triangle*, ▲) and *gpd2-ko*, *adh-ko* strain GDY15 (*filled diamond, ♦*) over 100 h in YPD medium. B) Production of glycerol (●) and ethanol (●) after 75h by WT, VSY0 and GDY15 strains. The experiment was performed in duplicates.


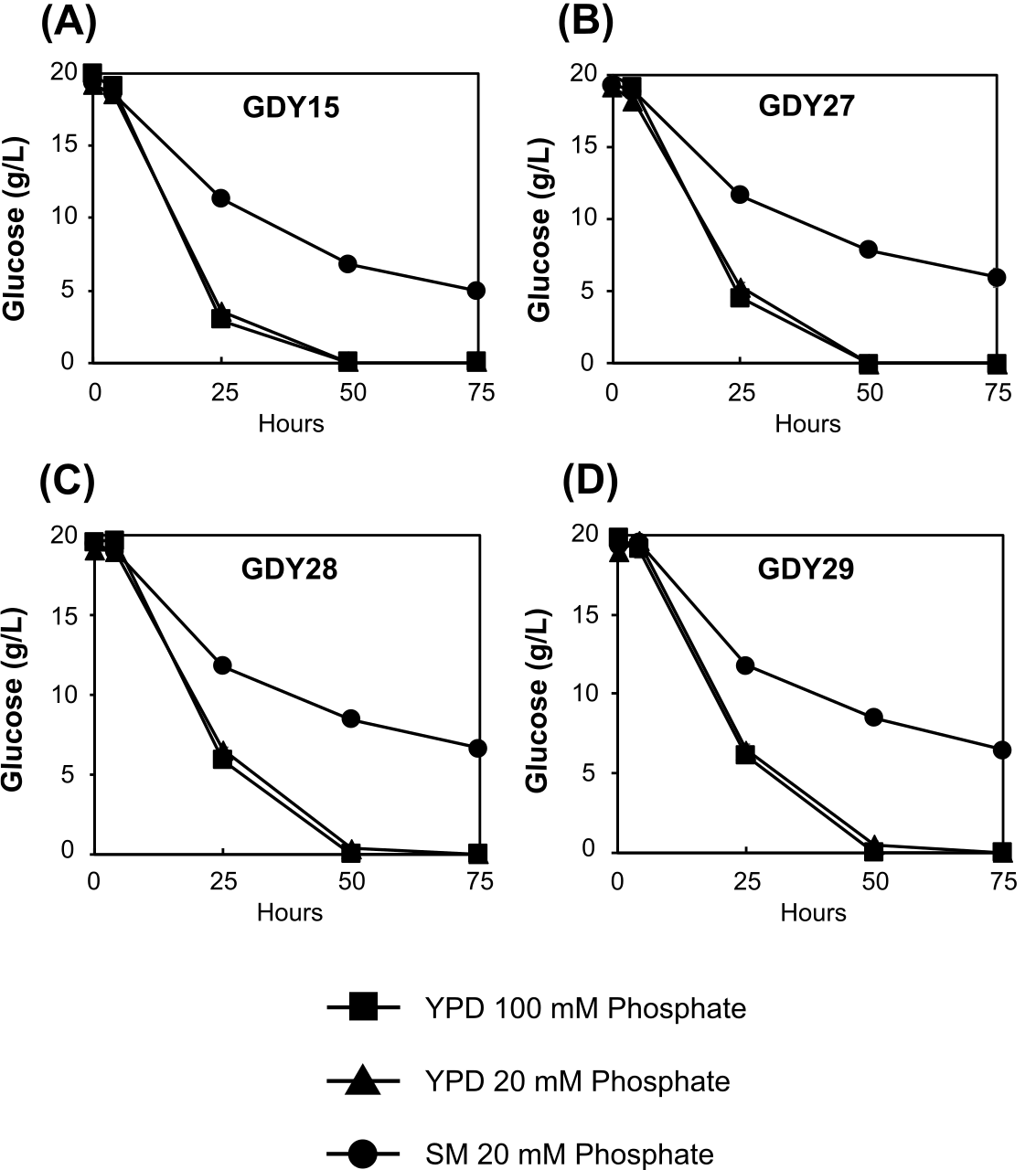


**Figure S2. Glucose consumption by *S. cerevisiae* strains with integrated reverse β-oxidation pathway variants in different cultivation media.** Glucose concentration (in g/L) by strains GDY15 (A), GDY27 (B) GDY28 (C) and GDY29 (D) in synthetic medium (SM) (*filled circle*, ●), YPD with 20 mM phosphate buffer (*filled triangle*, ▲) or YPD with 100 mM phosphate buffer (*filled square*, ■) over 75h. The experiment was performed in triplicates.


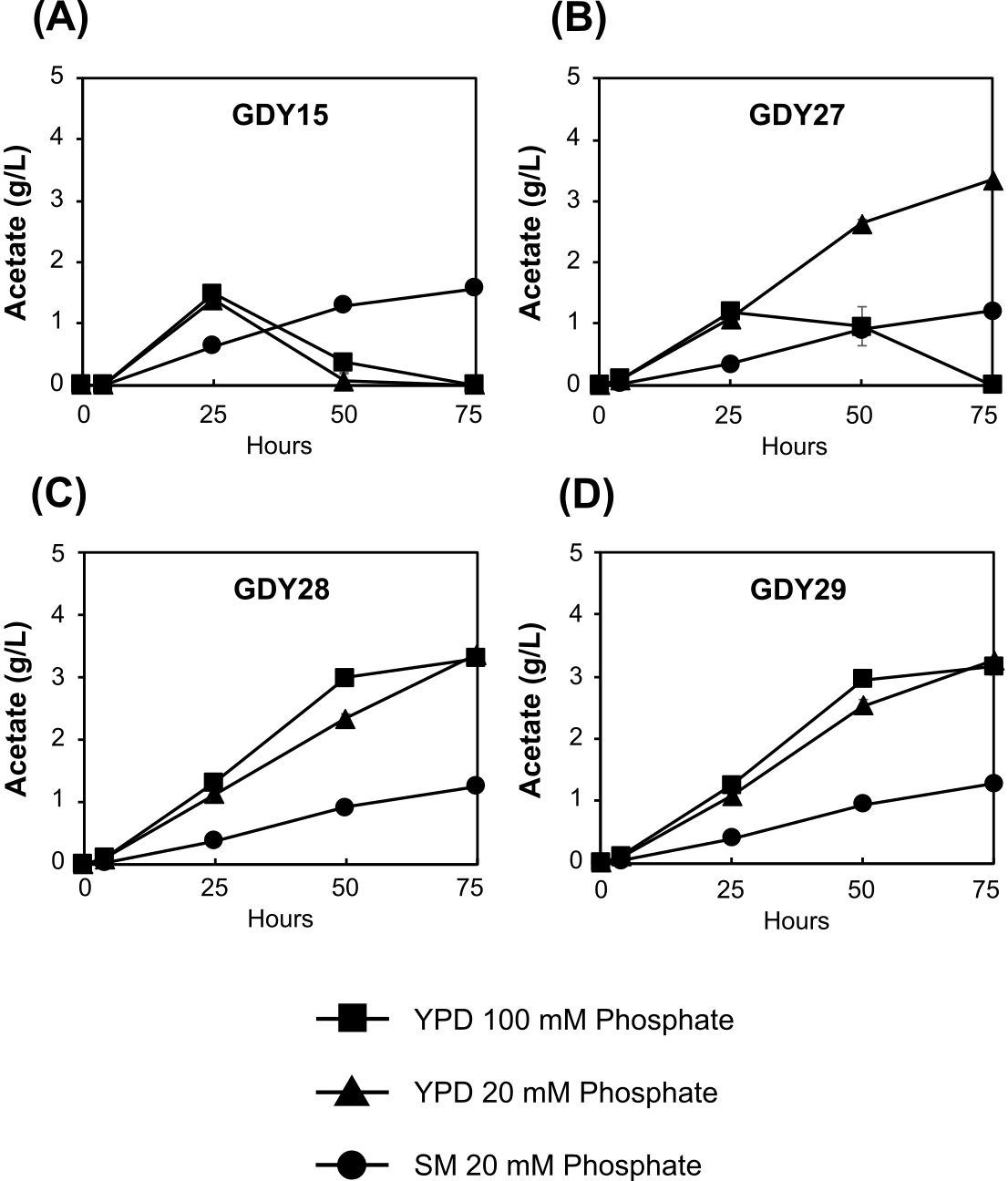


**Figure S3. Acetic acid production by *S. cerevisiae* strains with integrated reverse β-oxidation pathway variants in different cultivation media.** Acetic acid production (in g/L) by strains GDY15 (A), GDY27 (B) GDY28 (C) and GDY29 (D) in synthetic medium (SM) (*filled circle*, ●), YPD with 20 mM phosphate buffer (*filled triangle*, ▲) or YPD with 100 mM phosphate buffer (*filled square*, ■) over 75h. The experiment was performed in triplicates.
